# Early outcomes of pediatric heart transplantation: Impact of mechanical circulatory support and perioperative challenges. A single-center retrospective study

**DOI:** 10.62838/jccm-2026-0016

**Published:** 2026-04-30

**Authors:** Muhammad Shahzad, Reem Beheri, Bushra Algethami, Yasser Alheraish, Dimpna Albert, Felix W Tsai, Zohair Alhalees, Khaled Alarwan

**Affiliations:** King Faisal Specialist Hospital and Research Center Riyadh,Saudi Arabia

**Keywords:** pediatric heart failure, treatment, mechanical circulatory devices, transplantation, complications

## Abstract

**Background:**

Pediatric heart transplantation stays the gold standard treatment for end-stage heart failure, but outcomes are influenced by pre-transplant status and bridging strategies, particularly mechanical circulatory support (MCS).

**Objectives:**

To evaluate early outcomes following pediatric heart transplantation and assess the impact of pre-transplant MCS on survival and complications.

**Methods:**

We retrospectively analyzed all pediatric patients (<14 years) who underwent orthotopic heart transplantation at a single tertiary center between January 2020 and January 2025. Demographics, pre-transplant support, intraoperative data, and early outcomes were collected. Primary outcome was 30-day survival; secondary outcomes included acute rejection, infection, acute kidney injury (AKI), neurologic complications, and ICU/hospital length of stay (LOS). Comparative analyses were performed between patients having ECMO- and ventricular assist devises (VAD) using Fisher's exact and Wilcoxon rank-sum tests. Kaplan–Meier survival estimates were generated.

**Results:**

Thirty patients were transplanted (median age 9 years, 63.3% female). Most (96.7%) needed MCS, including 11 ECMO and 18 VAD. Thirty-day survival was 28/30 (93.5%, 95% CI 78–99). Acute rejection occurred in 3 (10%), infections in 4 (13.3%), AKI in 8 (26.7%), with two requiring CRRT, and neurologic complications in 3 (10.3%). Median ICU and hospital LOS were 20 and 37 days, respectively. ECMO patients had longer post-transplant ventilation (12 vs 6 days, p = 0.04) and ICU length of stay (LOS) compared to VAD patients. Total Ischemic time, and CPB times were associated with increased morbidity.

**Conclusions:**

Early outcomes after pediatric heart transplantation prove high short-term survival but substantial morbidity. ECMO bridging was associated with greater resource use than VAD. Improving donor heart ischemic time, donor-recipient matching, perioperative management, and early initiation of durable MCS may further improve outcomes.

## Introduction

Pediatric Heart failure (HF) is a clinical syndrome resulting from ventricular dysfunction. Pediatric patients with heart failure may experience prolonged waiting time until a suitable heart is available [1,2,3]. MCS devices such as extracorporeal membrane oxygenation (ECMO) and ventricular assist devices (VADs) are critical in stabilizing patients during this period by supporting organ perfusion and improving quality of life, and survival to transplant. Prolonged use of MCS devices can lead to complications, such as infection, thrombosis, and device failure [4].

Heart transplantation is the ultimate treatment of decompensated heart failure. In one study, heart failure-related intensive care unit hospitalizations in children with dilated cardiomyopathy (DCM) are increasing, and these children experience an in-hospital mortality of 11%, with a 30-day readmission rate after hospital discharge ranging from 13% to 34% [5].

Management of the post-operative heart transplantation is challenging, with an 8% 1-month mortality in contemporary cohorts [3]. Vast knowledge is needed to treat the hemodynamic instability, immunosuppression, and infectious complications (prevention and treatment) in addition to acute kidney injury and renal replacement therapy [6,7]. Specific attention should be paid to those who have VADs before the transplantation.

Although with the growing number of these patients who need mechanical circulatory support (MCS), such as ventricular assist devices (VADs) as a bridge to transplantation, the perioperative challenges have increased. Transplantation after MCS implantation introduces complex clinical scenarios. This analysis aims to highlight the clinical outcomes and challenges post heart transplantation on the native heart or following the MCS device implantation in pediatric patients.

The study aimed to evaluate early outcomes following pediatric heart transplantation and to assess the impact of pre-transplant mechanical circulatory support (MCS) on survival and complications.

## Methods

We performed a retrospective cohort study at a single tertiary center from January 2020 to January 2025, including all pediatric patients (<14 years) who underwent orthotopic heart transplantation. Patients with incomplete data, transplantation outside the Kingdom, or early mortality before ICU admission were excluded.

Data collection included demographics, diagnosis, comorbidities, pre-transplant support (ECMO, HeartMate 3, Berlin Heart EXCOR), ventilation status, and neurological history. Intraoperative variables included donor characteristics, ischemic time, aortic cross-clamp (ACC) time, cardiopulmonary bypass (CPB) duration, and complications (bleeding, instability, or ventricular dysfunction). The primary outcome was 30-day survival, while secondary outcomes include ICU and hospital length of stay, ventilation duration, acute rejection, infection, acute kidney injury, need for CRRT, liver dysfunction, and neurological complications.

Statistical analysis was conducted using SPSS. Continuous variables were reported as median [IQR] or mean ± SD, categorical variables as n (%). Fisher's exact or χ^2^ tests were used for categorical comparisons, and Wilcoxon rank-sum or *t*-tests for continuous outcomes. Proportions were presented with exact 95% confidence intervals (CIs); effect sizes were expressed as odds ratios (OR) with 95% CIs where possible. Linear regression assessed predictors of ICU LOS; logistic regression was used for binary outcomes with ≥10 events. Kaplan–Meier curves estimated 30-day survival, with log-rank testing for ECMO vs VAD. Analyses were considered exploratory given small sample size.

The study was approved by the Institutional Review Board, while the need for informed consent was waived.

## Results

### Patient demographics and clinical characteristics

A total of thirty pediatric patients who underwent heart transplantation were included. The median age at transplant was 9 years (IQR 6-12), with a female predominance of 19 (63.3%) patients. The median weight at transplant was 25.6 kg (IQR 22.3-35.0). The most common primary diagnosis was dilated cardiomyopathy (DCM), accounting for 20 (66.7%) patients. Complex congenital heart disease (CHD) was seen in 5 (16.7%) patients, while other cardiac conditions were seen in 5 (16.7%) patients. Table 1 summarizes the patients' demographics, primary diagnoses, and comorbidities.

**Table 1.** Patient demographics, primary diagnosis, and comorbidities (n=30)

**Variable**	**n (%), median [IQR]**
**Patient Demographics**	
Age at transplant, years	9 [6–12]
Female gender, n (%)	19 (63.3)
Male gender, n (%)	11 (36.6)
Weight at transplant, kg	25.6 [22.3–35.0]

**Primary Diagnosis**	
**DCM and related cases**	
- DCM, n (%)	20 (66.7)
**CHD and complex defects**	
- Complex CHD, n (%)	5 (16.7)
**Other cardiac conditions**	
- Complete Heart Block on PPM Post Arrest, n (%)	1 (3.3)
- Kawasaki Disease with Coronary Abnormalities, n (%)	1 (3.3)

VLCAD deficiency, n (%)	1 (3.3)
Familial hypercholesterolemia, n (%)	2 (6.6)

***DCM***: *Dilated cardiomyopathy;* ***CHD***: *Congenital heart disease;* ***IQR***: *Interquartile range.*

### Pre-transplant status and clinical parameters

The median duration of heart failure before transplant was 4 months (IQR 2-15). Most patients were critically ill before transplant, with 29 (96.7 %) requiring mechanical circulatory support. ECMO was used in 11 (36.6%) patients, HeartMate 3 in 8 (26.7%), and Berlin Heart EXCOR in 10 (33.3%). While 17 (56.7%) patients needed mechanical ventilation. The median duration of mechanical support was 26 days (IQR 6-92), and the median duration of pre-transplant ventilation was 8 days (IQR 4-15). Pre-heart transplant neurological deficits were documented in 5 (16.5%) patients, with ischemic injury being the most common (Table 2).

**Table 2.** Pre-transplant status and clinical parameters

**Variable**	**n (%), median [IQR]**
Duration of heart failure, months	04 [2–15]

**Decompensation status**	
- Mechanical support, n (%)	29 (96.7)
- Inotropic support, n (%)	01 (3.3)

**Mechanical support type**	
- ECMO, n (%)	11 (36.6)
- HeartMate 3, n (%)	08 (26.4)
- Berlin Heart EXCOR, n (%)	(33.3)
- Inotropic support only, n (%)	01 (3.3)

Duration of mechanical support before transplant, days	26 [6–92]
Pre-transplant ventilation, n (%)	17 (56.7)
Duration of ventilation, days	08 [2–15]

ECMO: Extracorporeal membrane oxygenation; VAD: Ventricular assist device; LVAD: Left ventricular assist device; ALT: Alanine aminotransferase; AST: Aspartate aminotransferase; CNS: Central nervous system; RSV: Respiratory syncytial virus; IQR: Interquartile range.

### Intraoperative data and complications

The median donor age was 28 years (IQR 13-36), with 17 (56.7%) male donors. The median ischemic time was 260 minutes (IQR 202-299). Intraoperative complications were common, with 17 (56.7%) patients experiencing significant bleeding (VAD or not, and on antiplatelets or not) and 12 (40.0%) with hemodynamic instability. The median aortic cross-clamp time was 185 minutes [IQR 102–218] and the median cardiopulmonary bypass (CPB) time was 231 minutes (IQR 206-301). Table 3 presents intraoperative data and complications.

**Table 3.** Intraoperative data and complications.

**Variable**	**n (%), median [IQR]**
Donor age, years	28 [13–36]

**Donor gender**	
- Male, n (%)	17 (56.7)
- Female, n (%)	13 (43.3)

Ischemic time, minutes	260 [202–299]

**Intraoperative complications**	
- Bleeding, n (%)	17 (56.7)
- Bleeding + Hypotension, n (%)	12 (40.0)
- RV low contractility + Hypotension, n (%)	1 (3.3)

ACC time, minutes	185 [102–218]
CPB time, minutes	231 [206–301]

ACC: Aortic cross clamp; CPB: Cardiopulmonary bypass; RV: Right ventricle; IQR: Interquartile range

### Early post-transplant outcomes (30 Days)

The 30-day survival rate was 28 (93.5%) patients. Acute graft rejection occurred in 3 (9.9%) patients, with varied severity. Most rejections were managed by immunosuppressive therapy adjustments, high-dose steroids, or intravenous immunoglobulin (IVIG). Post-transplant infections developed in 4 (13.2%) patients (in whom donors were not infected), while acute kidney injury occurred in 8 (26.7%) patients, with 2 (6.7%) requiring continuous renal replacement therapy (CRRT). Four (13.2%) patients received antibiotics, as donors were infected. Liver dysfunction was seen in 6 (19%) patients, who improved with no chronicity. Neurological complications were reported in 3 (10.3%) patients. Among Three (10%) patients who developed neurological insult after heart transplant, one of them recovered completely, and two had a remaining neurological deficit with limb weakness. The median duration of post-operative ventilation was 8 days (IQR 2-15), ICU stay was 20 days (IQR 11-33), and total hospital stay was 37 days (IQR 22-66). Table 4 summarizes early post-transplant outcomes.

**Table 4.** Early post-transplant outcomes (30 days)

**Variable**	**n (%), median [IQR]**
30-day survival, n (%)	**28 (93.5)**
Acute graft rejection, n (%)	3 (9)

**Rejection**	
- Cellular rejection, steroids/Tac optimization, n (%)	1 (3.3)
- Cellular/Humoral rejection, unspecified, n (%)	1 (3.3)
- Humoral rejection, IVIG^b^/plasmapheresis n (%)	1 (3.3)

Infections within 30 days, n (%)	4 (13.3)
Acute kidney injury, n (%)	8 (26.7)
CRRT, n (%)	2 (6.7)
Liver dysfunction, n (%)	6 (19.8)
Neurological complications, n (%)	3 (10.3)
Post-op ventilation duration, days	8 [2–15]
ICU LOS, days	20 [11–33]
Hospital LOS, days	37 [22–66]

IVIG: Intravenous immunoglobulin; FK: Tacrolimus (FK506); ICU: Intensive care unit; IQR: Interquartile range; LOS: Length of stay.

a Severity of rejection or infection;

b Management approach

All transplanted patients required postoperative admission to the intensive care unit (ICU). The median ICU length of stay was 20 days (IQR 11–33), reflecting the high acuity of illness at transplantation. Mechanical ventilation was required in all patients immediately postoperatively, with a median ventilation duration of 8 days (IQR 2–15). ECMO-bridged patients experienced a significantly longer duration of postoperative ventilation compared with VAD-bridged patients (12 vs 6 days, p = 0.04).

Vasoactive support was frequently required in the early postoperative period, particularly in patients with prolonged CPB and ischemic times. Renal dysfunction and fluid overload were common contributors to prolonged ICU stay, with 26.7% developing acute kidney injury and 6.7% requiring continuous renal replacement therapy. Neurological complications, when present, were associated with delayed extubation and longer ICU courses.

### Comparative analyses

ECMO-supported patients had significantly longer post-operative ventilation (median 12 vs 6 days, *p* = 0.04). ICU and hospital LOS trended longer in ECMO-supported patients but did not reach significance. Pre-transplant neurologic deficits were associated with a higher risk of post-transplant deficits (40% vs 4%, *p* = 0.03, Fisher's exact).

Table 5 compares early post-transplant outcomes in patients bridged with ECMO, n = 11 versus ventricular assist devices (VAD: HeartMate 3 or Berlin Heart EXCOR, n = 18). Survival at 30 days was comparable between groups (91% vs 94%).

**Table 5.** comparative analysis of the heart transplant after ECMO or VAD support.

**Variable**	**ECMO (n = 11)**	**VAD (n = 18)**
30-day survival	10/11 (91%)	17/18 (94%)
Acute rejection	1 (9%)	2 (11%)
Infections	2 (18%)	2 (11%)
Acute kidney injury (AKI)	4 (36%)	4 (22%)
CRRT required	1 (9%)	1 (6%)
Neurological complications	2 (18%)	1 (6%)
Liver dysfunction	2 (18%)	4 (22%)
Post-op ventilation (days)	12 [9–16]	6 [5–9]
ICU LOS (days)	24 [18–34]	16 [10–24]
Hospital LOS (days)	42 [28–66]	34 [22–50]

Abbreviations: ECMO = extracorporeal membrane oxygenation; VAD = ventricular assist device (HeartMate 3, Berlin Heart EXCOR); CRRT = continuous renal replacement therapy; LOS = length of stay.

### Exploratory regression analyses

**Figure 1.**
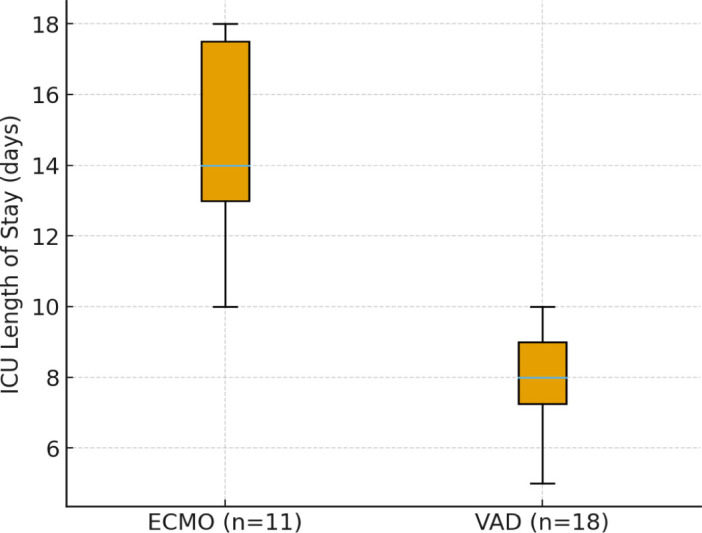
**ICU length of stay (LOS) stratified by pre-transplant support type.** Boxplot comparing ICU LOS in patients bridged with extracorporeal membrane oxygenation (ECMO, n = 11) versus ventricular assist devices (VAD: HeartMate 3 and Berlin Heart EXCOR, n = 18). Median ICU LOS was longer in ECMO patients (12 days, IQR 9–16) compared to VAD patients (6 days, IQR 5–9), p = 0.04 (Wilcoxon rank-sum test). Boxes represent the interquartile range, whiskers the range, and horizontal lines the median.

**Fig. 2.**
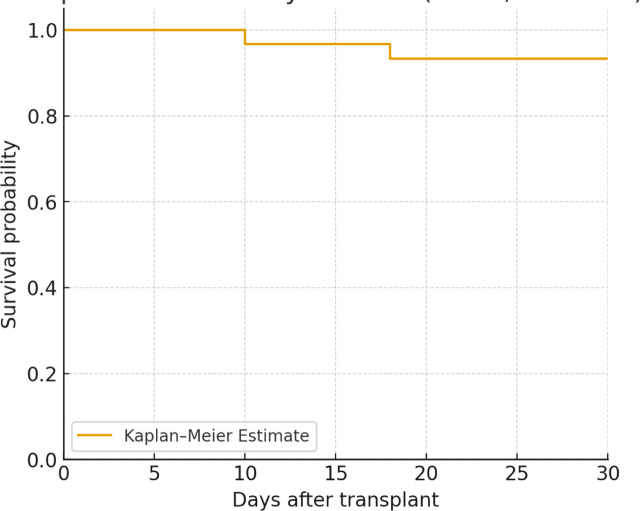
**Kaplan–Meier 30-day survival after pediatric heart transplantation (n = 30).** Kaplan–Meier survival curve demonstrates 30-day survival of 28/30 patients (93.5%, 95% CI 78–99). Two deaths occurred on days 10 and 18. Survival probability remained stable after day 18. Due to only two events, regression analysis was not performed.

Each 30-minute increase in ischemic time was associated with higher odds of AKI (OR 1.4, 95% CI 1.0–2.1, *p* = 0.07). CPB duration correlated with longer ICU stay (β = 0.11 days per minute, 95% CI 0.02–0.20, *p* = 0.02). After adjusting for MCS type, CPB time stayed an independent predictor of ICU LOS (β = 0.09, 95% CI 0.01–0.18, *p* = 0.03). Logistic regression for 30-day survival could not be reliably performed due to only two deaths, producing unstable estimates (Figure 1 and 2).

## Discussion

In data analysis of thirty pediatric heart transplantation recipients, extensive demographics were described, followed by details of clinical presentations, intraoperative course, and early post-transplant outcomes. Pediatric heart transplantation is still one of the most lifesaving, yet challenging interventions for end-stage heart failure from many cardiac etiologies. This analysis highlighted the findings that can be used for critical reflection in patient management and outcomes of pediatric transplant practice.

In our analysis, the median age at transplantation was 9 years, and more females (63.3%) than males were transplanted. In previous studies, demographic distributions have been variable, but some researchers observed the same gender predominance [8]. In contrast to what has been reported in this study, the significant female predominance here does not seem to be a common phenomenon and is worth investigating in a larger cohort to identify potential biological or social determinants of increased referral or indication for transplantation in females.

Two-thirds of our patients (66.7%) were diagnosed with dilated cardiomyopathy (DCM), the primary diagnosis. This fits well with the global data that DCM is the most common indication for pediatric heart transplantation [9]. Other less frequent diagnoses, e.g., complex congenital heart diseases (16.7%) showed the variation in disease presentation. However, representation of CHD does warrant attention, because cases of CHD are often complicated by earlier surgical intervention, and anatomical complexity, which may in turn affect both operative and post-operative outcomes [10]. In another study, eighty-nine patients were supported. The type of support was left ventricular assist devices in eighty-six patients (97%), and biventricular support in three patients (3%). Age and weight at support were 2.8 ± 0.5 years (46 days to 13 years 2 months), and 9.2 ± 0.1kg. Diagnosis of the patients included dilated cardiomyopathy in 74 (83%), congenital heart diseases in 8 (9%), restrictive cardiomyopathy in 5 (5%), and ischemic heart diseases in two patients (2%). [11]

Indeed, we often see an infrequent occurrence of important comorbidities such as epilepsy or autoimmune diseases (both 3.3%), as selection tends to favor candidates with fewer systemic problems. However, it must be remembered that even rare comorbid conditions can affect management decisions and outcomes [12].

Heart failure prior to transplantation was short (median 4 months), and hence the disease was likely to be progressing rapidly, leading to the need for transplantation. Nearly all patients (96.7%) were critically ill and required mechanical circulatory support before transplantation. This data concurs with the available literature, where pediatric recipients are commonly found to be in an urgent and unstable clinical state [13]. Mechanical circulatory support, including ECMO or VAD, has become indispensable in stabilizing critically ill pediatric patients awaiting heart transplantation, dramatically improving pretransplant survival. Our cohort shows that MCS has been extensively used to bridge critically ill patients to transplantation.

However, prolonged mechanical support periods, with a median duration of 26 days, carry risks such as infection and neurological complications, which were prevalent in our study 13.2% infections and 10.3% neurological deficits [14]. As these complications demonstrate the double edge of prolonged MCS, close monitoring protocols must be conducted to care for the prevention and early detection of infections and neurologic deficits [15].

Although prolonged, these results are within acceptable ranges, with a median ischemic time of 260 minutes and a median CPB duration of 231 minutes. This cohort shares a correlation between extended ischemic and CPB times and increased magnitude of post-operative complications, including AKI, bleeding, and neurologic deficits. Increased donor ischemic time may be attributed to a long travel distance in the Gulf region. [16] Furthermore, there is a need for improved intraoperative bleeding control measures in patients with significant bleeding as suggested by the fact that 56.7% of such patients have significant bleeding [17].

In addition, prolonged intraoperative times require better and quicker preoperative recipient matching, harvesting, as well as logistical optimization to reduce ischemic injury to the donor heart. Due to the limited availability of donors, it became difficult to precisely match the donor and recipient status. So, SCOT (Saudi Center for Organ Transplantation) is working to reduce this risk. However, the implications of donor to recipient age mismatch should be further studied, as the median donor age was 28 years, and donors were male (56.7%). These factors affect the outcomes in pediatric recipients of adult donor hearts [18].

One of our key findings is the 93.5% 30-day survival rate, which is like international benchmarks [19]. However, post-transplant immunosuppression remains overly complex, given the early complications, including acute graft rejection in 9% of patients. This substantial risk may reflect the differences in donor-recipient age (median age recipients 9; median age donors 28). Increasing pediatric donor availability is a priority. In our cohort, the median donor age (28 years) exceeded the median recipient age (9 years), raising concerns about donor–recipient mismatch and its potential impact on outcomes. Tacrolimus adjustments and intravenous immunoglobulin (IVIG) administration were important facets in acute rejection management through optimization of immunosuppressive therapy. With support from literature, one must recognize the need for individualized, aggressive immunosuppressive regimens in pediatric transplantation to decrease rejection episodes, but side effects must also be closely monitored [20].

Infections after transplant persist as a significant problem, affecting almost half the cohort (26.7%). Infection control thus becomes a major concern and requires strict hygiene protocols, adequate surveillance, as well as early antimicrobial therapy if infection is suspected [21]. Similarly, AKI has been seen to occur frequently (26.7%) with a need for CRRT in 2 (6.7) % cases, as was observed in prior studies that have associated the length of CPB and ischemic time with renal dysfunction [22]. Preoperative renal protective measures, careful fluid management, medicine dose adjustments, and early dialysis are among the proactive measures that may reduce the incidence and severity of AKI [23].

The complicated interplay between pre-transplant neurological deficits, prolonged MCS, operative duration, and immunosuppressive drug neurotoxicity was again reflected in the fact that 10.3% of patients suffered neurological complications in the postoperative period [24].

These findings highlight the need for neurologic monitoring in regular intervals and early post-operative neuroimaging studies after transplantation for early diagnoses and management of complications. In the long-term follow-up, one patient recovered, and two patients remained on neurological follow-up, one for seizure control and another for hemiplegia. We are planning to conduct another comprehensive study to describe the neurological complications in these patients.

In our cohort, extended ICU stays and hospitalizations (median 20 and 37 days, respectively) indicate that our patients were severely ill, suffered complications, and had a prolonged recovery process, as is expected for pediatric heart transplant patients [25]. The prolonged ICU stay observed in this cohort underscores the significant critical care burden associated with pediatric heart transplantation, particularly in patients bridged with mechanical circulatory support. ECMO-bridged patients demonstrated a more complex postoperative course, characterized by longer ventilation duration and extended ICU stays, reflecting greater pre-transplant instability and higher perioperative risk. These findings align with existing literature describing ECMO as an effective but resource-intensive bridge to transplantation. Our patients had a critical pre-operative course, with complications of Berlin Heart EXCOR and HeartMate 3 that prolonged the post-heart transplant ICU stay. We need to better understand the importance of the initiation of heart failure treatment at the primary health level.

### Study limitations and future directions

Although this study offered many insights, being retrospective in nature, having a small cohort size and being designed at a single center, limited generalizability. Further work is needed in the form of future multicenter collaborative studies with larger patient cohorts to strengthen findings and allow more robust statistical analysis. However, further research is also needed to understand the impact of pediatric heart transplantation for these patients, including quality of life and psychosocial implications, which should include prospective studies examining long-term outcomes.

In addition, further investigation of optimal immunosuppressive protocols, new mechanical support technologies, and means of reducing ischemic and bypass time could improve outcomes. Personalized medicine pathways in pediatric heart transplantation may be provided by genetic studies to study susceptibility to rejection, infections, or drug toxicity [26,27].

### Clinical implications and recommendations:

Some important aspects of pediatric heart transplantation improvement include:

Providing enhanced preoperative assessment and stabilization with earlier initiation of MCS when indicated to limit organ dysfunction.
Improved intraoperative management strategies to reduce ischemic, CPB and renal injury times as well as bleeding.
Postoperative robust infection control protocols to minimize immunosuppression related infections.
Use of tailored immunosuppressive regimens tailored according to individual risk profile and careful surveillance for rejection, infection, and drug adverse effects.
Targeted rehabilitation and backing treatments managing to launch early movement, enhance sustenance and psychological backing, which potentially could shorten hospital stays and improve personal satisfaction.

Overall, this study proves how children are confronted with many challenges in pediatric heart transplantation and identifies key areas for focused clinical attention and research. Finally, there is promise of much better outcomes and better quality of life for pediatric transplant recipients through continuous improvement in the pursuit of rigorous evidence-based practice and collaborative research for improving pediatric transplant management.

## Conclusion

Pediatric heart transplantation in our center achieved excellent early survival despite most children being critically ill and dependent on mechanical circulatory support. However, postoperative morbidity and ICU resource utilization remained substantial, particularly among ECMO-bridged patients. Longer ischemic and cardiopulmonary bypass times were associated with increased complications and prolonged ICU stay. These findings highlight the fragile balance between life-saving intervention and intensive postoperative recovery in this population. Optimizing pre-transplant stabilization, donor–recipient logistics, and perioperative care is essential to improve both survival and the quality of early recovery.
